# Malaria Related Perceptions, Care Seeking after Onset of Fever and Anti-Malarial Drug Use in Malaria Endemic Settings of Southwest Ethiopia

**DOI:** 10.1371/journal.pone.0160234

**Published:** 2016-08-12

**Authors:** Zewdie Birhanu, Lakew Abebe, Morankar Sudhakar, Gunawardena Dissanayake, Yemane Ye-ebiyo Yihdego, Guda Alemayehu, Delenasaw Yewhalaw

**Affiliations:** 1 Department of Health Education and Behavioral Sciences, College of Health Sciences, Jimma University, Jimma, Ethiopia; 2 President’s Malaria Initiative, United States Agency for International Development, Addis Ababa, Ethiopia; 3 Abt Associates, Africa Indoor Residual Spraying, Accra, Ghana; 4 Department of Medical Laboratory Sciences and Pathology, College of Health Sciences, Jimma University, Jimma, Ethiopia; 5 Tropical and Infectious Diseases Research Center, Jimma University, Jimma, Ethiopia; Centro de Pesquisas Rene Rachou, BRAZIL

## Abstract

**Background:**

Prompt care seeking and appropriate use of anti-malarial drugs are critical components of malaria prevention and control. This study assessed malaria related perceptions, care seeking behavior and anti-malarial drug use in malaria endemic settings of Ethiopia.

**Methods:**

Data were generated from a community based cross-sectional study conducted among 798 households during January 2014 as part of a larger household behavioral study in three malaria endemic districts of Jimma Zone, Southwest Ethiopia. Both quantitative and qualitative data were collected and analyzed using SPSS 17.0 and STATA 12.0.

**Results:**

In this study, only 76.1% of the respondents associated malaria to mosquito bite, and incorrect beliefs and perceptions were noted. Despite moderate level of knowledge (estimated mean = 62.2, Std Err = 0.7, 95% CI: 60.6–63.8%), quite high favorable attitude (overall estimated mean = 91.5, Std Err = 0.6, 95% CI: 90.1–92.9%) were recorded towards malaria preventive measures. The mean attitude score for prompt care seeking, appropriate use of anti-malarial drugs, LLIN use and Indoor Residual Spray acceptance was 98.5 (Std Err = 0.4, 95% CI:97.5–99.4), 92.7 (Std Err = 0.6 95% CI:91.5–93.9), 88.8 (Std Err = 0.5, 95% CI:85.5–92.1) and 86.5 (Std Err = 1.2, 95% CI: 83.9–89.1), respectively. The prevalence of fever was 2.9% (116/4107) and of the study participants with fever, 71.9% (95% CI: 65.5–78.3%) sought care and all of them consulted formal health care system. However, only 17 (19.8%) sought care within 24 hours after onset of fever. The frequency of care seeking was higher (77.8%, n = 21/27) and more prompt (28.6%, 6/21) for children under five as compared to old age groups despite it was not statistically significant (p > 0.05). However, higher median time of seeking first care was observed among Muslims and people who did not attend school (p < 0.05). Of those who used anti-malarial drugs, 9.1% indicated that they used it inappropriately through saving and/or sharing. Irregular availability of anti-malarial drugs; irregular presence of frontline health workers and misconceptions were mentioned to contribute to delayed care seeking and irrational use of anti-malarial drugs.

**Conclusions:**

Although care seeking behavior for febrile illness was quite high in this community, the habit of prompt care seeking was very limited. Thus, malaria prevention and control programs need to take into account local misconceptions and wrong perceptions, and health system factors to achieve optimal health seeking behavior in such malaria endemic settings.

## Background

Malaria is a life threatening disease and one of the major public health and socio-economic problems globally [1]. According to the World Health Organization (WHO) report, malaria was responsible for 198 million illnesses worldwide leading to about 584, 000 deaths in 2014. Africa carries the highest burden with 90% of all malaria deaths [1]. In 2013, an estimated 437, 000 African children died due to malaria before their fifth birthday [1]. Malaria also remains an important public health and socio-economic challenge in Ethiopia-an estimated 60% (about 50 million) of the population were at risk of contracting malaria in 2014 [2]. In 2013/2014, 2,627,182 laboratory confirmed malaria cases were reported in Ethiopia where Oromia Region was the third largest contributor with 474, 641 cases [3]. This latest report indicated that there is a declining trend in overall national burden although an increasing trend was observed in some regions [3]. In the same year (2013/2014), malaria (confirmed with P. falciparum) was the fifth cause of morbidity (5.3%) and the fifth leading cause of health facility admission (2.4%) (confirmed with P. falciparum alone) in Ethiopia. Likewise, it was the seventh (2.5%) leading cause of health facility admission among children under five [4].

The Action and Investment to defeat Malaria (AIM) 2016–2030 strategy underscored that malaria is not only a health issue, but also a broader developmental, socio-political, economic, environmental, agricultural, educational, biological and social issue [5]. This strategy laid strong emphasis on the importance of keeping target community at the center of the fight against malaria and highlights the need for inclusive and collaborative efforts to create a malaria-free world by 2030 [5]. As a result of this and other earlier [6] and recent global efforts such as President Malaria Initiative Strategy (2015–2020) which reaffirmed the long term goal of worldwide malaria eradications [7], malaria prevention and control has received substantial attention in Ethiopia [8,9]. This is because the government committed itself to eliminate malaria within specific geographical areas with historically low malaria transmission and to achieve near-zero malaria death in the remaining malaria-endemic areas of the country in 2015 [8]. The plan has aimed to achieve 100% treatment seeking within 24 hours of onset of fever; 100% diagnosis using Rapid Diagnostic Tests (RDTs) and/or microscopy of malaria suspected cases; and 100% anti-malarial drug use if found positive as per the national guideline [8]. Consequently, the government of Ethiopia has been making progressive efforts to sustain malaria control and move towards the pre-elimination and elimination phase [8,9,10]. Access to malaria diagnosis has been decentralized to community level health facilities through the introduction of RDTs [9, 10]. Even though microscopy is the sole technique being used to diagnose malaria in hospitals, health centers and higher private facilities in Ethiopia, RDT is being used for diagnosis of malaria, at community level, by health extension workers [8–12]. The current national malaria guideline recommends that all malaria suspected cases should be diagnosed using RDTs and /or microscopy in any health facilities including private care providers [8,11,12]. However, clinical or symptoms based malaria diagnosis and treatment is still practiced in Ethiopia [4]. Nevertheless, the supply of RDTs is restricted to public health facilities which cannot support diagnosis by microscopy in remote areas without electricity [12]. Furthermore, the government updated the 2011–2015 national health sector strategic plan to 2014–2020 to scale up the fight against malaria through increasing community participation and mobilization [11]. Most importantly, the updated plan has given substantial attention to early diagnosis and effective treatment of malaria cases and intends to implement evidence based effective malaria prevention and control interventions [11].

Early recognition and reporting for malaria diagnosis as well as seeking effective treatment of malaria and appropriate use of anti-malarial drugs are critical elements of malaria control program. These play a crucial role in the move towards elimination and achieving near zero malaria death [13,14]. WHO strongly recommends that in malaria endemic areas every suspected malaria case must be tested and every confirmed case must be treated with quality-assured anti-malarial drugs [13, 14]. Currently, artemisinin-based combination therapy (ACT) is being used to treat all clinically and parasitologically diagnosed uncomplicated P. *falciparum* malaria in Ethiopia and it is widely available at all levels of public health facilities in the country but commonly distributed by the Health Extension Workers (HEWs) at the community level [12]. Nevertheless, the distribution of ACT is restricted to public health facilities. On the other hand, oral Chloroquine is a first line drug in Ethiopia for all malaria patients with P. vivax, and it is available in all health facilities including privately owned ones [12]. Primaquine and oral quinine are also being used in Ethiopia (at health center and hospital levels) depending on the patients’ conditions and nature of infections [12]. On the other hand, seeking care promptly after onset of illness is vital for effective management of malaria [14]. This is because early treatment ensures rapid and complete elimination of the parasite from the patient’s blood which in turn prevents progression of the disease to severe disease or death, and to chronic infection that leads to malaria-related anemia [15]. Most importantly, early treatment reduces transmission by reducing the infectious reservoir, it and prevents the emergence and spread of resistance to anti-malarial medicines [15]. However, prompt and effective identification of malaria cases depends on the recognition of the sign and symptoms of malaria and prompt health care seeking behavior to appropriate health care facilities to receive prompt treatment [14]. Some earlier studies documented that the rate of seeking prompt care in Ethiopia is low [16–18]. Thus, given the prospect of malaria elimination and eventual eradication, it is imperative to continually evaluate community perceptions and practice of care seeking in malaria endemic settings of Ethiopia to support the move towards the set targets. Therefore, this investigation presents malaria related knowledge, care seeking behavior after onset of fever and anti-malaria drug use among households in selected districts of Jimma Zone, Ethiopia.

## Methods and Materials

The data were part of a community based cross sectional study conducted as part of a larger household behavioral survey in three districts (Mana, Gomma and Kersa) of Jimma Zone, Oromia Region, Ethiopia, during Dec 2013 to Jan 2014. The household behavioral survey was conducted to have baseline information for malaria education interventions implemented through school students and religious leaders in the selected districts. This baseline study assessed community perceptions towards malaria, use of long lasting insecticide treated nets, care seeking behaviors for fever/malaria, anti-malarial drug use, attitude and acceptance of indoor residual spraying. However, this analysis was restricted to malaria related perceptions, care seeking and anti-malarial drug use. The detailed survey method has been described elsewhere [19]. In brief, the data were collected from 798 selected households in three study districts. The sample size was determined using single population proportion formula (n = (Z 1-α/_2_)^2^ p (1-p)/ d^2^) based on the following assumptions: Proportion of under five children who slept under insecticide treated bed net the previous night is 55.4% [16], 5% marginal error, 95% confidence interval, design effect of 2 and 10% non-response rate. Since the study was conducted as part of a larger study on household malaria preventive behaviors, the sample size computation was based on use of insecticide treated net. Based on probability proportional to population size, the sample size was allocated to each district and thirteen Gandas (smallest administrative unit in Oromia, Ethiopia) with high malaria risk were randomly selected in the three districts (4 from Kersa, 4 from Mana and 5 from Gomma). Within district, the sample size was proportionally allocated to each selected Gandas based on the total number of households. Finally, the households were systematically selected in each Ganda using a random start point at the center of Ganda by spinning a pen. In each Ganda, the sampling interval was determined by dividing the total number of households to the allocated sample size. The initial household was selected randomly between the first household (at the center of Ganda) and the sampling interval. The subsequent households were identified by adding the sampling interval to the previous household number until the required sample was obtained. Heads of the households were interviewed during the survey. However, if the household head was not present at the time of the visit, the spouse was interviewed.

To explore some barriers, perceptions and misconceptions related to malaria, care seeking and anti-malarial drug use, six Focus Group Discussions (FGDs) and eleven Key Informant Interviews (KIIs) were simultaneously conducted with purposively selected community members, health workers, teachers and religious leaders. FGD participants were married males and females who lived in the study community for longer durations. Thus, males were head of households and female participants were mothers. The participants were purposively recruited with the primary aim to include information-rich respondents on malaria and its prevention methods. In addition, respondents were also included based on some socio-demographic background such as age, education and religion to capture diverse views. Each FGD consisted of 7 to 11 participants. On the other hand, key informants were purposively selected for the study taking into account their experience in malaria control program (e.g. health workers). Similarly, religious leaders and school teachers were selected for the interview since the project had been using them as a change agent in malaria education intervention in the selected districts and villages.

### Measurements

Data were collected using standard questionnaire adapted from relevant literatures such as malaria indicator survey and health and demographic survey [16, 17] to assess care seeking behavior for fever, knowledge and attitude related to malaria and its prevention and control. Except knowledge related items where respondents were probed once, all the questions were unprompted type. Four main questions related to cause of malaria, sign and symptoms, prevention measures and vulnerable groups were used to assess respondents’ knowledge on malaria. The four major questions contained ten specific items which included three signs and symptoms of malaria (i.e. fever, feeling cold/shivering and headache), mosquito bite as cause of malaria, four prevention measures (sleeping under insecticide treated net, spray house with insecticide, cleaning surroundings areas, and filling poodles/draining stagnant water), and knowledge of vulnerable groups to malaria infections (pregnant women, and children under five). For each item, correct answer was assigned ‘1’ and incorrect answer as ‘0’. Then, respondents’ knowledge was computed by summing up all correct responses. The higher the score the higher the knowledge level it indicates. To check for the association between knowledge and median time of care seeking, the overall knowledge score was categorized as low (0–50), moderate (51–70) and high (71–100). Likewise, attitude towards malaria prevention measures were assessed using seventeen items scored on three point ordinal scale ranging from disagree (1) to agree (3). The items covered insecticide treated net use (5 items), timely care seeking (2 items), proper use of anti-malarial drugs (5 items) and acceptance for indoor residual spray (5 items). To compute the overall attitude score, the items were summed up and finally converted to 100%. The higher the score the higher the favorable attitude towards malaria prevention measures it indicates.

With respect to measures related to care seeking behavior, fourteen items were used to assess care seeking behavior and anti-malarial drug use. The items were designed in such a way to capture important factors in care seeking for fever including time of care seeking, source of care, and anti-malaria drug use. Early treatment seeking behavior was defined as seeking help or advice from health facilities within 24 hours of onset of fever. Occurrence of fever among household members was measured within two weeks recall period immediately preceding the survey. Respondents (head of household or spouse) provided information on households’ members’ malaria episodes. Head of the household/spouse were chosen to provide information about family members as they deemed to be the most knowledgeable in the family about family health. Respondents were shown all anti-malarial drugs to help them recall which anti-malarial drug they had taken during the two weeks period prior to the interview date and reduce recall bias. In this study, appropriate use of anti-malaria drugs was defined as taking complete dose without mal-practices such as discontinuations, sharing with others, saving at home for latter use, skipping doses, taking over and /or under recommended dosage at a time

### Data collection Methods

The quantitative data were collected by experienced and trained field enumerators. The tool was translated into Afan Oromo (local language) and pretested on 5% of the sample size in similar settings. The whole process of data collection was closely supervised by the investigators. Each FGD was moderated by two experienced public health specialists with master’s degree qualifications who were well conversant to the local language, Afan Oromo. However, each key informant interview was conducted by a single interviewer. The FGDs and Key informant interviews were audio-recorded to supplement the notes taken during the interview.

### Data processing and analysis

The data were analyzed using SPSS 17.0 and STATA 12.0. Descriptive statistics were used to present the findings. Using the survey design variables (i.e. Ganda as first stage sampling units within districts and households as second stage units), the dataset was declared as survey data. Estimated means and proportions, with its respective confidence intervals, were computed using the survey data. Knowledge and attitude score was classified as low, moderate and high according to their percentiles. In addition, non-parametric Kernel density estimate was employed to fit into malaria related knowledge and attitude score. Non-parametric local polynomial regression smooth with second degree was applied to estimate impact of age on care seeking. The results were presented using narrative text, tables and graphs. Each FGD and interview data were transcribed verbatim and then translated into English language by moderators for open coding process. Further analysis was done manually based on thematic analysis approach. However, the analysis was made by the investigators and the findings were triangulated with the quantitative results.

### Ethical consideration

The study was reviewed and approved by Jimma University Ethics Review Committee (Ref. No: RPGC/260/2013. Respondents were provided with detail information about the study through a consent process and informed verbal consent was obtained from all respondents. Then consent was documented for each respondent and kept at Jimma University. The consent form and consent procedures were also reviewed and approved by Jimma University Ethics Review Committee. The study involved very minimal risk coupled with anonymity. Through permission from ethics review committee, verbal consent form was used with each respondent.

## Results

### Demographic characteristics of the respondents

Overall, 798 households with 4,107 household members living in the surveyed households were included during the analysis. Household members who experienced fever during the last two weeks prior to the survey date were constituted the sample size for care seeking. Detailed background information of the participants who actually responded to the questionnaire was presented elsewhere [16]. The majority of the respondents (83.6%) were from rural areas, and half (50.0%) of them did not read and write. The majority (80.2%) of the respondents were Muslims. Detail information about respondents’ background characteristics can be obtained from S1 File.

### Awareness and knowledge related to causation of malaria

Malaria is known as ‘Busa’ and ‘Woba’ in the locality in Afan Oromo and Amharic, respectively. Table 1 presents respondents’ awareness and knowledge malaria. The study revealed that knowledge about classical signs and symptoms of malaria was very high: chilling (87.3%), fever or hot body (74.1%) and headache (73.1%) were the most frequently mentioned signs and symptoms of malaria. The study also revealed that community members often define malaria illness in terms of fever, headache or chilling. With respective to causal explanation, 602 (75.4%) knew that mosquito bite causes malaria. Likewise, most FGD participants also had good awareness about the cause of malaria even to the extent of differentiating female anopheles mosquitoes as transmitting disease causing agent.

*“…*.*malaria is transmitted by mosquito bite*, *if the female mosquito bites someone who has malaria and if that mosquito bites other person*, *it transmits the disease*.*”*

[36 years old Male, FGD participant]

“…*People get malaria form mosquito*. *When a mosquito bites someone*, *it becomes a disease in the body and causes fever*, *chills*, *headache and loss of appetite*. *Finally*, *it becomes a serious disease”*

[28 years old female, FGD participant who did not attend school]

**Table 1:** Perceptions and knowledge of respondents related to malaria, Jimma, Ethiopia (Jan 2014).

Variables	Frequency	%
**Sign and symptoms of malaria**		
Chilling	697	87.3
Fever/hot body	591	74.1
Headache	583	73.1
Nausea and vomit	268	33.6
body weakness	264	33.1
Loss of appetite	259	32.5
body ache or joint pain	184	23.1
Others (pale eye, dizziness, diarrhea, back pain)	112	14
**Perceived causes of malaria**		
Mosquito bite	602	75.4
Unhygienic food/drink	350	43.9
Cold or changing weather	163	20.4
Hunger	145	18.2
Getting soaked with rain	117	14.7
Don't know	26	3.3
Others*	30	7.1
**Perception on how to prevent malaria**		
Keeping house surrounding’s clean	611	76.6
Sleeping under mosquito net	607	76.1
Filling puddles	419	52.5
Not drinking/eating dirty water/food	213	26.7
House spraying with insecticides	83	10.4
Others**	76	9.6
Don't know	14	1.8
**Perceived vulnerable groups to malaria infections**		
Children under five	696	87.2
Pregnant women	622	77.9
Elders	88	11
Adult female	39	4.9
Adult male	22	2.8
Don’t know	34	4.3

* Eating maize, eating sugarcane, *Witchcraft*

** don’t get soaked with rain, *eating garlic*, *not consuming sugarcane*, *using Vaseline*, *drink alcohol*

However, there were also misperceptions by few respondents regarding the role of mosquito in malaria transmission. In one male FGD, even though the majority of the participants supported that mosquito bite causes malaria, few participants strongly argued that malaria is caused by a kick or an attack by the devil which often hides itself in swampy area.

*“…The cause of malaria is Jinni [Devil] and it is known since ages; our elders told us a lot about it*. *In this nearby forest*, *there is devil and it speaks out but you can’t see it; sometimes it orders you not to do activities*. *If you breach its order*, *immediately you will be getting sick and die within nine days*. *I have never seen when mosquito causes malaria*!*”*

[42 years old male, FGD participant]

Similarly, there were also other several misconceptions and wrong beliefs held by some respondents. Eating unhygienic food and/or drinking unclean water (43.9%), cold or changing weather condition (20.4%), hunger (18.2%) and getting soaked with rain (14.7%) were cited as causes malaria. In women FGDs, few participants associated malaria with lack of hygiene and poor sanitations.

*“People get malaria due to poor hygiene; not separating human house from animals*, *failure to have separate houses for dining and for food preparation; presence of containers which store water in residential area”*

[29 years old female, FGD].

### Knowledge related to malaria prevention and control

The majority of the respondents (76.6%) mentioned that keeping living area clean, sleeping under mosquito net (76.1%) and filling puddles/pits (52.5%) prevent malaria. House spraying with chemical insecticides was perceived as prevention method by few (10.4%) respondents. Many FGD participants, both in male and female FGDs, also mentioned that keeping personal and environmental hygiene and use of latrine prevent malaria infection.

***“****We can prevent malaria by keeping our environment*, *our body and our children’s body clean; by draining of stagnant water; avoiding swampy area and using latrine and keeping it clean*.*”*

[37 years Male, FGD participants]. Few respondents also had some misconceptions on how to prevent malaria (Table 1).

### Attitude towards malaria preventive methods

Most respondents had quite high favorable attitude towards malaria preventive measures. Attitude towards seeking prompt care was exceptionally favorable (estimated mean = 98.5 (Std Err = 0.4, 95% CI: 97.5–99.4). In the same way, the mean estimation for attitude towards appropriate use of anti-malarial drugs, LLIN use and Indoor Residual Spraying acceptance were 92.7 (Std Err = 0.6, 95% CI:91.5–93.9), 88.8 (Std Err = 0.5, 95% CI:85.5–92.1) and 86.5 (Std Err = 1.2, 95% CI:83.9–89.1), respectively. Item based analysis showed that 28.9% of the respondents supported sharing anti-malarial drugs with others; 27.1% did not believe on preventive role of IRS, and 23.4% of the respondents showed less willingness to cooperate during house spraying. The qualitative data revealed that various factors contributed to negative attitude towards IRS such as lack of clear information about the spray, doubt on the efficacy of the chemical insecticides being sprayed, perceptions that chemical insecticides eventually increases reproduction of other insects, and disappointed with exhaustive work to move household utensils before and after the spray.

“Some people may refuse due to lack of knowledge and hating its odor and ugly color deposited on the wall.”

[32 years old male, FGD]

*“There are some people who are not willing to spray their home because they believe that it increases abundance of other insects after few months*.*”*

[32 years old female, FGD]

**Fig 1:**
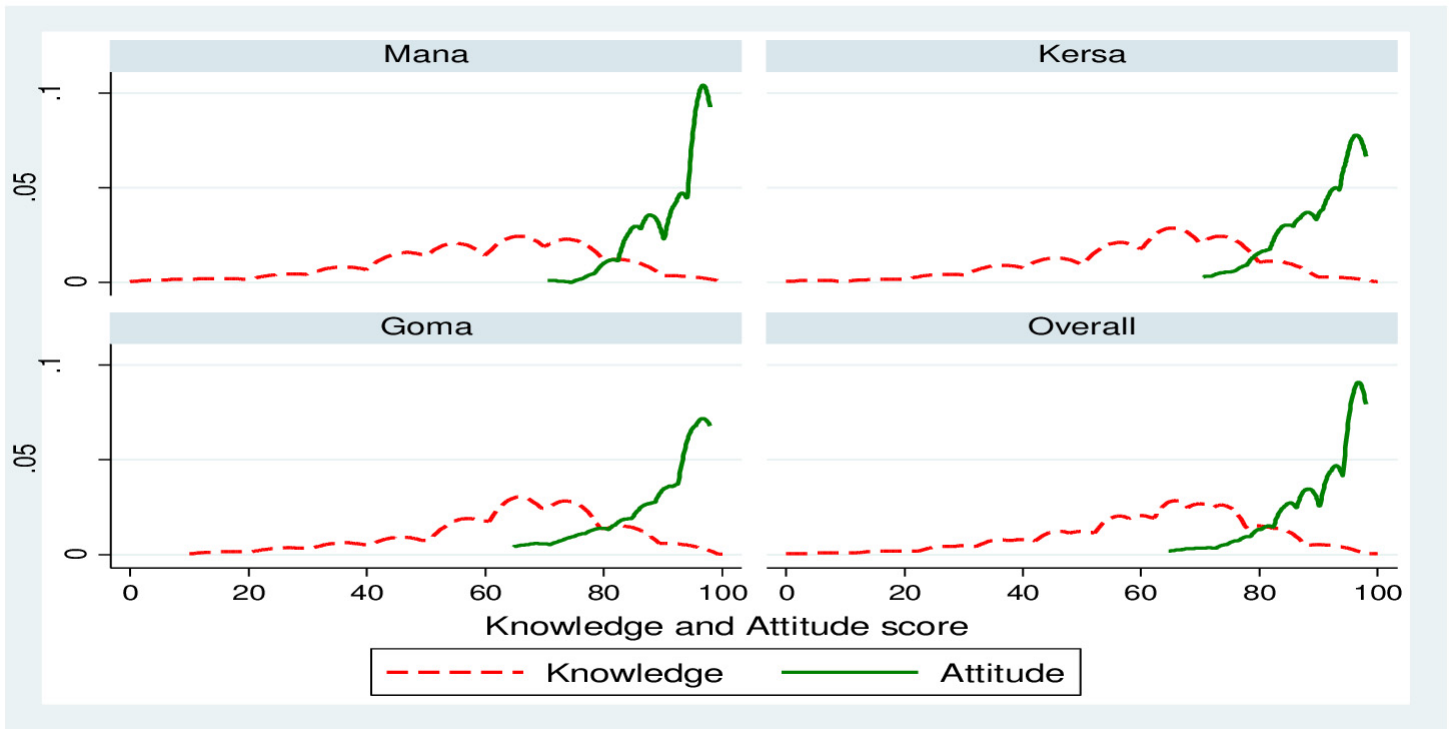
Kernel density estimates for malaria related knowledge and attitude scores by district, Jimma, Jan 2014. Overall, the density of knowledge score was lower as compared to the attitude score and in each district similar pattern was observed. This means despite low level of knowledge, the majority of the respondents had quite high favorable attitude towards malaria preventive measures (overall estimated mean = 91.5, Std Err = 0.6, 95% CI: 90.1–92.9%) towards malaria preventive measures. Generally, no single respondent scored less than 60% on the attitude scale. In contrast, there were respondents who had low level of knowledge (estimated mean = 62.2, Std Err = 0.7, 95% CI: 60.6–63.8%) on malaria and its preventive measures.

The study revealed that 26.9% (95% CI: 22.4–31.5%), 50.8% (95% CI: 42.7–58.7%) and 22.3% (95% CI: 16.2–28.3%) of the respondents scored low, moderate and high level of malaria related knowledge, respectively. Fig 1 shows Kernel density estimate for overall knowledge and attitude by district after the score was adjusted to 100%. S1 Dataset contains data on respondents’ knowledge and attitude towards malaria prevention and control measures.

### History of fever among household members

The study showed that one hundred sixteen (estimated proportion = 2.9%, 95% CI: 0.6–5.2%) of the household members included during house-to house interview had fever two weeks immediately preceding the survey. Table 2 presents reported prevalence of fever by district and place of residence. The prevalence of fever was less varying by district and place of residence although relatively higher among rural residents and in Gomma district. More than half (54.3%) of the fever episodes were experienced by females. A little more than a quarter (28.4%) of the fever episodes were experienced by children below age of five (data not shown). Supporting information on prevalence of fever can be accessed from S2 Dataset.

**Table 2:** Reported prevalence of fever two weeks prior to the survey, Jimma, Ethiopia (Jan 2014).

Background characteristics		N	Household members with fever episodes	Estimated prevalence of fever (%)	95% CI (%)
District	Kersa	1450	42	2.9	0.5–14.4
	Mana	1108	22	2.1	0.6–7.1
Place of Residence	Gomma	1549	52	3.3	0.9–10.4
	Urban	667	12	1.1	0.3–4.7
	Rural	3440	104	3.3	1.5–7.1
LLIN use during the last 15 days	Yes	1535	40	2.4	1.1–3.4
	No	2572	76	3.1	1.2–7.8
**Overall estimated prevalence of fever**		**4107**	**116**	**2.9**	**0.6–5.2**

### Care seeking after onset of fever

**Fig 2:**
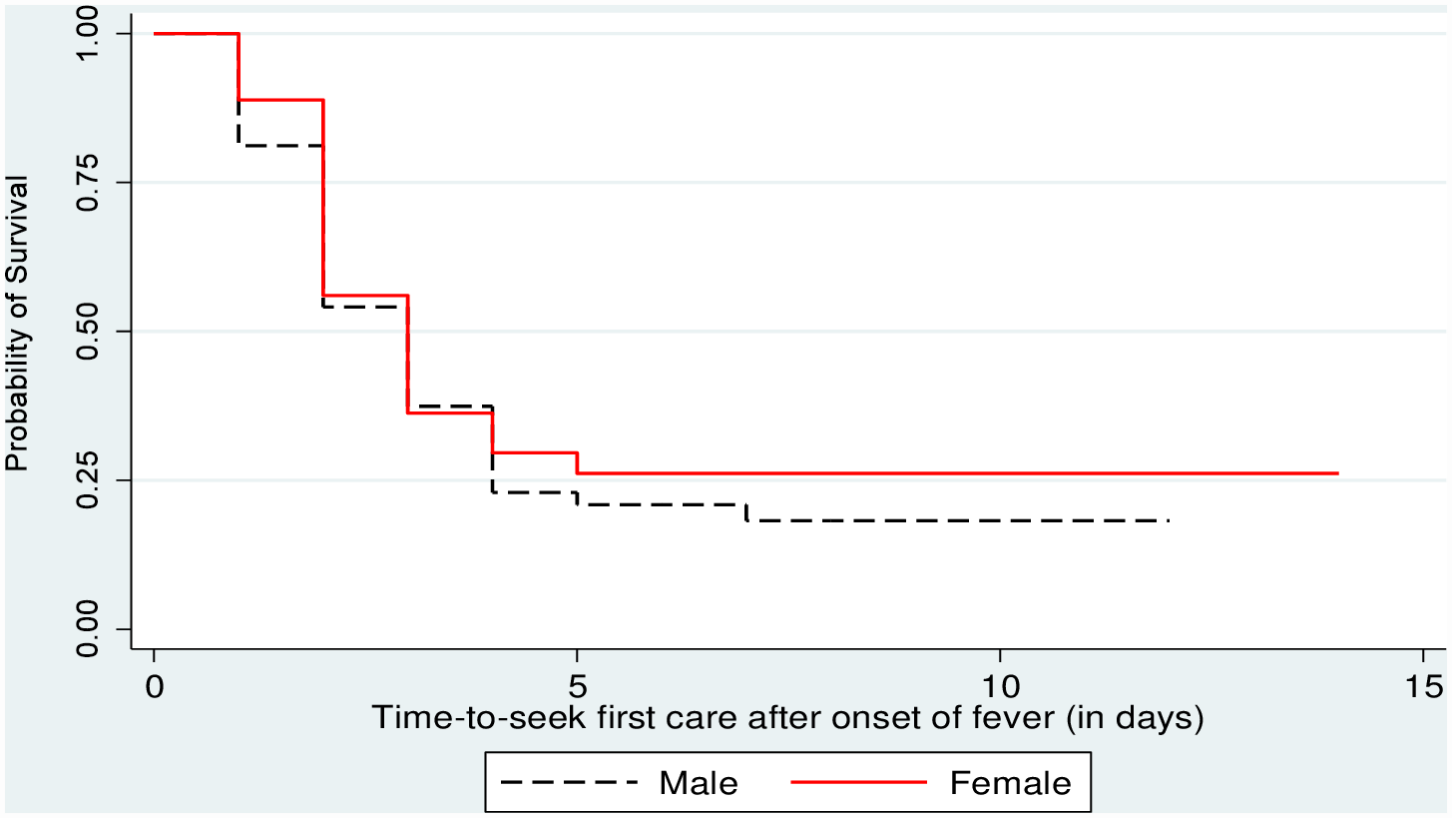
Kaplan-Meier Survival function for time-to-seek first care after onset of fever by sex, Jimma, Jan 2014. As indicated in the figure, females had longer survival time to seek first care after onset of fever although the difference was not statistically significant (log Rank X^2^ = 0.667; P = 0.414). The mean survival time was 4.3 (Std Err = 0.5, 95%CI: 3.2–5.4) and 5.4 (Std Err = 0.6, 95%CI: 4.2–6.8) days for males and females, respectively. The overall estimated mean survival time was 5.1 (Std Err = 0.5, 95% CI: 4.2–6.0).

Of those 116 household members who experienced fever, 71.9% (95% CI: 65.5–78.3%), ever sought some sort of care. S3 Dataset provides supplementary information on care seeking practices by the study participants. Fig 2 shows Kaplan-Meier survival curve for time–to-seek first care after onset of fever by sex. Although a large portion, of febrile individuals sought care, only 17(19.8%) of them sought the first care within 24 hours after onset of fever. The mean and median time of seeking first care was 2.5 (Std Err = 0.1) days and 2.00 days, respectively. Table 3 shows the result of non-parametric tests for median time difference (in days) for care seeking by selected background characteristics. Consequently, statistically significant difference in median time of seeking first care was recorded only for religion and education. Statistically significant median time difference for seeking first care was recorded for Muslims and people who did not attend school (p < 0.05).

**Table 3:** Mann-Whitney (U) and Kruskal Wallis (H) tests for median time difference for seeking first care after onset of fever, Jimma, Ethiopia.

Characteristics	Category	Median time to seek care (min, max)	U/H, p-value
Residence	Urban	2 (1,4)	401.50, 0.897
	Rural	2 (1, 7)	
Sex	Male	2 (1,7)	913.00, 0.932
	Female	2 (1,5)	
Religion	Muslim	2 (1,7)	215.00, 0.021
	Christian	2 (1,3)	
Respondent education	Not attended school	2 (1,7)	349.50,0.029
	Attended school	2 (1,3)	
Spouse education	No formal education	2 (1,7)	431.00, 0.206
	Formal education	2 (1,3)	
Family size	1–5	2 (1,7)	913.00, 0.974
	> = 6	2 (2,5)	
District	Kersa	2 (1,4)	0.634, 0.739
	Mana	2 (1.7)	
	Goma	2 (1,5)	
Respondents’ Knowledge	High	2 (1,5)	0.793,0.673
	Moderate	2 (1,7)	
	Low	2.5(1,5)	
Caretakers’ occupation	Farmers	2 (1,7)	2.909, 0.256
	Private job	3(1,3)	
	Others*	2 (1,4)	
Age in years	≤5yrs	2 (1,5)	1.172,0.568
	6–18 yrs	2 (1,7)	
	>18 yrs)	2 (1,5)	

* merchant, private employ, government employee

### Pattern of care seeking after onset of fever

**Fig 3:**
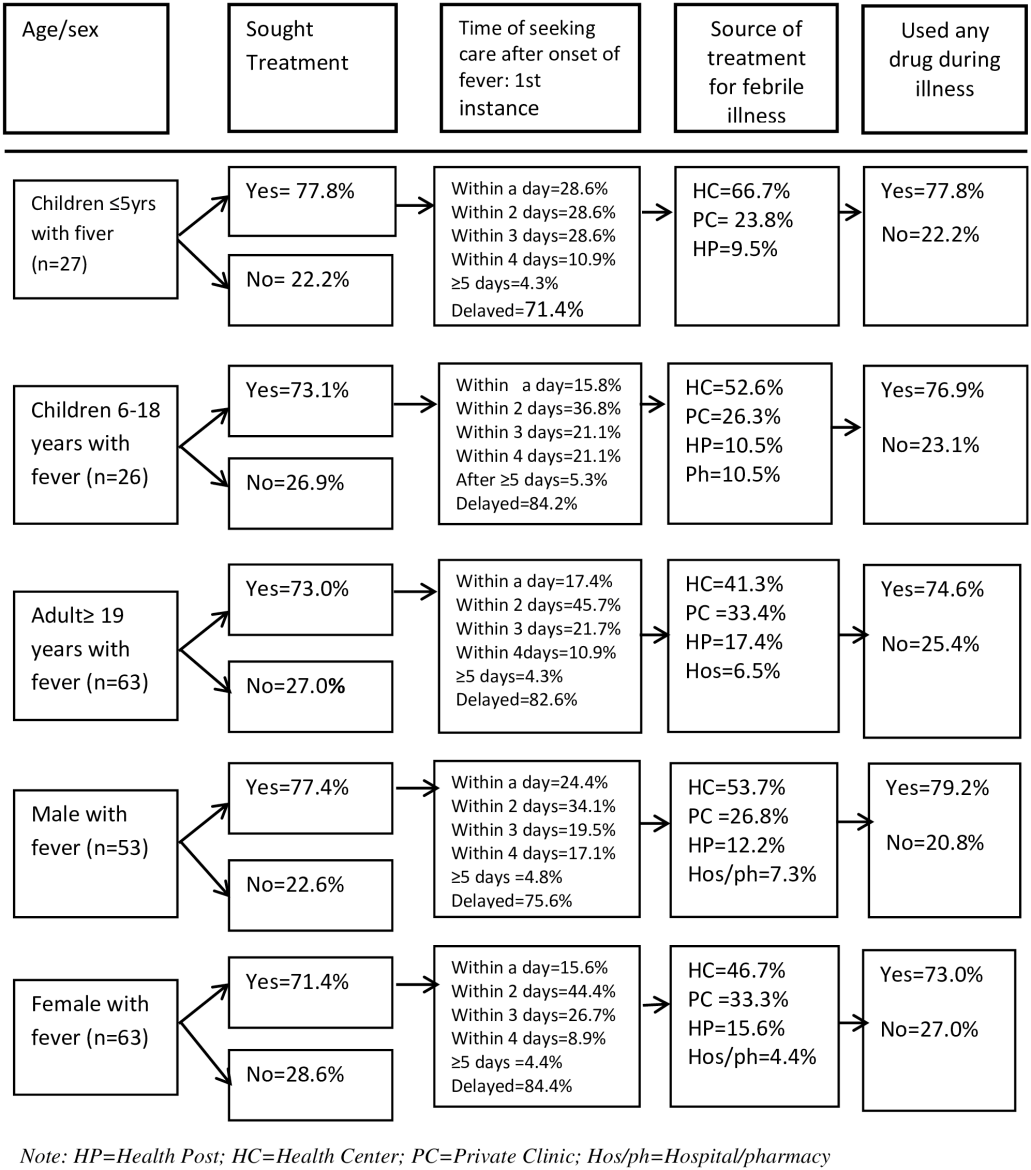
Pattern of seeking care after onset of fever by age and sex category, Jimma, Jan 2014. The frequency of seeking any care for fever was almost similar across age groups even though slightly higher for children under five (77.8%) as compared to other age groups. However, there was a slight gender disparity (male 77.4% versus female 71.0%). On the other hand, the frequency of seeking prompt care was relatively higher for children below the age five (28.6%) and lower for older children (age 6–18 years old) (15.8%). Likewise, more males (24.4%) were promptly taken to health facilities than females (15.6%) within a day. The frequency of using public health facilities tended to decline with age while the tendency of using private clinics increased with age which means that respondents tended to visit public health facilities for childhood fever whereas private clinics were chosen for adulthood fever (private clinic: 23.8% for children versus 33.4% for adults).

**Fig 4:**
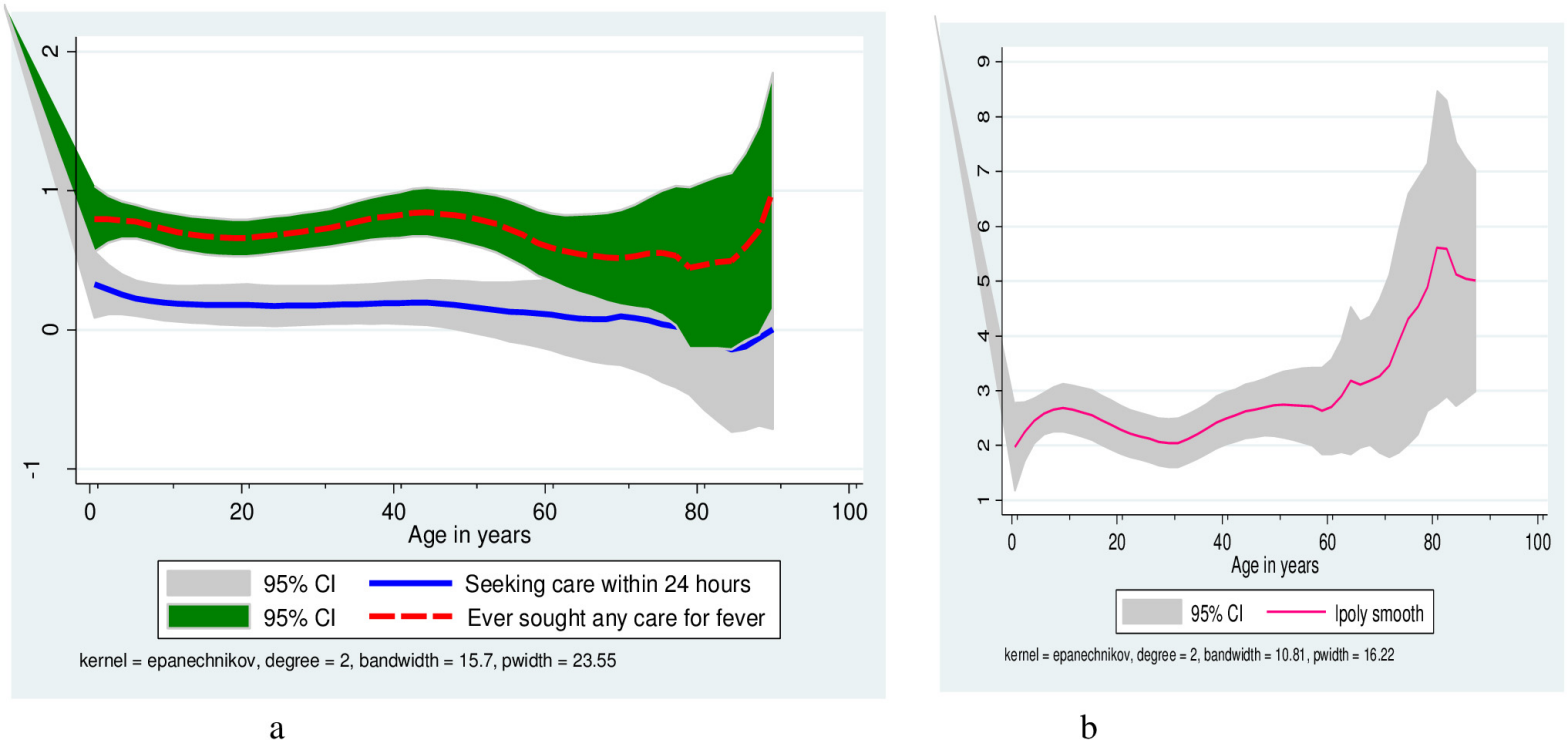
Local polynomial regression smooth for probability of seeking any care, and prompt among people with fever, Jimma, Jan 2014. “Fig 4a” shows the probability of seeking any care for fever (red-broken line) and how prompt it is (i.e. within 24 hours after onset of fever) (blue-solid line). Accordingly, in both cases the probability of seeking any care for fever and seeking care within 24 hours are higher during early ages. “Fig 4b” indicates time to seek first care (in days) by age and it is also more prompt for childhood illnesses.

Fig 3 presents pattern of care seeking after onset of fever by age and sex. However, there was no statistically significant difference in median of seeking first care by gender. The probability of seeking care for fever was predicted using non-parametric local polynomial regression. Fig 4 shows local polynomial regression smooth for probability of seeking any care, and prompt care by age. In the qualitative evidence, most respondents mentioned that these days, people seek care for fever quickly. Nevertheless, when asked to report what quick care seeking mean to them, most of them cited that prompt care seeking is after two to three days of onset of fever. The habit of care seeking was more prompt if the illness was perceived as severe and slow if it seemed less severe.

*“…*..*Most of the time people seek care after two days because they wait until they learn whether the illness gets serious or not*.*”*

[Religious leader, Key informant].

### Reported reasons for delayed care seeking for fever

#### Unavailability of HEWs

Most FGDs participants mentioned that irregular availability of HEWs discouraged people from timely seeking care at health post.

#### Unavailability of anti-malarial drugs

There was high concern among respondents that anti-malarial drugs are hardly available at local public health facilities. *“…Most of the time they refer us to buy drugs from the private clinics which is more expensive for people like us or farmers*.*”* [31 years old female, FGD discussant]

#### Perception of severity of the illness

To investigate how severe the illness is, some respondents mentioned that some people tended to wait and see for some days.

#### Lack of resources

Due to scarcity of resources such as money for transportation and other expenses, people tended to seek care late especially if they are far away from health facilities.

### Source of care

Half of those who sought care visited health center, and nearly one third (30.2%) of the respondents used private health clinics (Table 4). None of the respondents reported visiting traditional sources of care for fever. It was mentioned that there was an increased awareness about malaria and people knew that malaria is treated only at health facilitities. Nevertheless, few respondents in the qualitative part of the study mentioned that there were families who still tended to depend on spiritual remedies (eg. pray to God, visit holly water in case of severe malaria) even though the majority went for health facility. In this regard, it was documented that some families still prefer to visit traditional healers as sources of care and resort to formal health facilities when traditional treatment fails.

*“…*.*some people first go to traditional healers for every problem but when it becomes serious they decide to go to health facilities”*.

[22 years female, FGD participant]

**Table 4:** Treatment seeking behavior after onset of fever among respondents, Jimma, Ethiopia (Jan 2014).

Variables		Frequency	Percent
Where did you seek advice or treatment (n = 86)	Health center	43	50.0
	Private clinic	26	30.2
	Health post	12	14.0
	Hospital/pharmacy	5	5.8
How many days after the fever began did first seek advice or treatment	24 hour	17	19.8
	2 days	34	39.5
	3 days	20	23.3
	≥4 days	15	17.4
Shared drug with others (n = 88)	Yes	8	9.1
	No	80	90.9
Diagnosed for fever before drug use (n = 88)	Yes	53	60.2
	No	35	39.8

### Anti-malarial drug use

**Fig 5:**
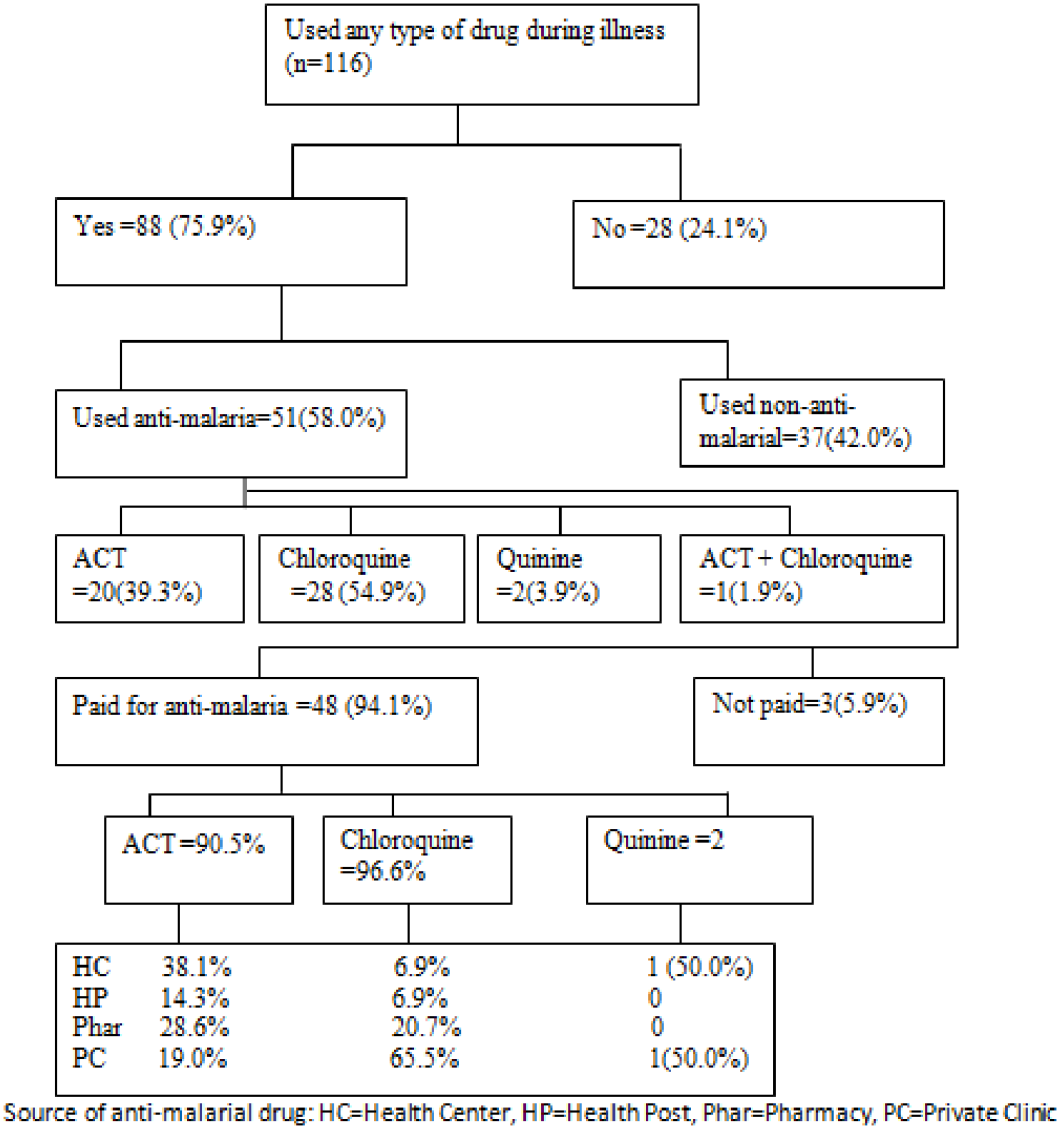
Anti-malarial drug use amonspeople with fever, Jimma. Jan2014. Of the household members who reported having fever, 88 (75.9%) used certain type of drug. However, only 51 (58.0%) used anti-malaria drugs and the remaining used non-anti-malarial drugs such as antipyretics, antibiotic and others. Of those who received anti-malarial drugs, 28(54.9%) received Chloroquine followed by ACT which accounted for 20 (39.3%). The majority (94.1%) of those who used anti-malarial drugs reported that they paid for it. Private clinics (45.1%), pharmacies (23.5%) and health centers (21.6%) constituted the major source of anti-malarial drugs. Only (9.8%) of those who used anti-malarial drugs obtained it from community health posts.

Detail of anti-malaria drug use is presented on Fig 5. Despite the majority of those who received any drugs for fever reported appropriate use, 8(9.1%) disclosed that they shared it with someone else (Table 4). In the qualitative report, most respondents mentioned that even though most people properly use anti-malarial drugs, there are people who did not comply with the instructions given by health workers. These mentioned various forms of misuse included, taking more tablets at a time than recommended (i.e. to get quick relief), discontinuing once felt better, missing tablets (e.g. due to being busy with household chores and field activities and sour taste of tablet) and sharing and saving drugs for future use. According to the qualitative data, several factors contributed to misuse of anti-malarial drugs, particularly for sharing and/or saving. These included irregular availability of HEWs, lack of proper advice by health workers, shortage of drugs at local health facilities, increased awareness about malaria and its treatment options, increased tendency of expecting ACT for every fever and headache and availability of ACT in private clinics and pharmacies.

*“Some people buy this drug and keep it in their home to use in the future*, *in case another member of the family becomes ill*.*”*[Health worker, key informant]. Another participant also mentioned supporting statement “…*if we identify the sickness as malaria*, *we can share the drugs until the person gets treatment*

[48 years old male, FGD participant].

## Discussion

Early and effective treatment of malaria is a critical element in malaria control programs as it prevents the progression of the disease to a severe one, reduces malaria related morbidity and mortality and reduces the overall parasite reservoir in the community [8, 13, 14]. However, early treatment depends upon prompt recognition of sign and symptoms of malaria in the household and prompt self-referral plus availability and access to appropriate health care facilities. In this study, consistent with the findings of many previous reports [18, 20–23], awareness about signs and symptoms of malaria was quite high: about three-fourth of the respondents were aware of the classical signs and symptoms of malaria (feeling cold, fever and headache) and knew that mosquito bite causes malaria. This recognition of malaria symptoms could have an implication for early consultation at appropriate health care facilities and correct treatment of malaria. The overall knowledge level was encouraging compared to some previous studies [20, 22]. However, consistent with the findings of some earlier studies [20, 21, 24, 25], there had still misconceptions and wrong beliefs on causation and prevention measures of malaria. In some cases, malaria was linked to less hygienic food or drinks and also to maize or sugarcane, witchcraft or devils attack, cold weather conditions, hunger and getting soaked with rain. Such belief would have a negative influence on prompt care seeking and treatment sources preferences [14, 20]. However, the impact of such beliefs was not observed in the current study as visiting non-formal sources of care were non-existent. However, this study explored the experience of few respondents with febrile illness which might not exemplify the normative care seeking habits in the community.

On the other hand, even though the majority of the respondents knew that malaria is caused by mosquito bite, some tended to associate malaria infection with poor hygiene and sanitation. The attribution of malaria to poor hygiene and sanitation might be due to over emphasizing the role of environment and hygiene in the cause and prevention of many communicable diseases including malaria. Despite a quarter of participants had misconceptions about the cause of malaria, most respondents had favorable attitude towards malaria preventive measures reflecting that people could have favorable attitude without having sufficient knowledge about the basics of malaria and its prevention measures. This might be due to the historical malaria messaging approach in that it primarily focuses on creating motivations than providing clear facts and information about the disease and how to prevent it. It must be noted that behavior change that would occur in such a way may not be long-lasting and is easily susceptible to relapse due to lack of strength to withstand counter influences [26] calling for the need to improve the community’s correct knowledge on malaria. Of course, some significant proportion of the respondents had negative attitude towards IRS which was mainly linked to lack of awareness and poor management practice of spray operations as noted in the qualitative part of the study.

In many endemic areas, malaria is the major cause of fever that gives an approximate idea of the amount of malaria in a given community [27]. However, people with partial immunity often do not develop fever in spite of the presence of malaria parasites in the blood. In addition, fever may not also be present in individuals where erythrocytic schizogony has been interrupted but where the gametocytes are still present [27]. In this study, the prevalence of fever was 2.8% which is much lower compared to some previous local studies [16, 18] and studies done elsewhere [24, 28, 29]. The fact that this study was conducted during the dry season can partially explain the lower prevalence of fever which can also be attributed to the real reduction of malaria in the community in recent years.

In this study, nearly three-fourth of those who experienced fever had sought care which is similar with some previous findings [18, 21, 22, 28] but a better figure compared to some other studies [25, 29, 30]. In fact, there was also a study that reported higher frequency of care seeking for fever [24]. The habit of care seeking was relatively higher for under five children implying that there is increasing concern and sense of urgency for childhood illness among parents. However, the frequency of care seeking was lower for females. This indicates gender disparity in accessing treatment and care for malaria in the community. Thus, understanding of the gender related dynamics of treatment-seeking behavior and decision-making process within households are crucial to ensure effective malaria control interventions.

The critical finding in this study is that only one in five of the febrile individuals sought care within the recommended time period. Despite high awareness of fever as a symptom of malaria, seeking treatment for fever tended to be delayed for a number of days, especially unacceptably slow for older children (i.e. aged 6–18 years) and females. Some previous studies also reported similar observations [18, 21, 22] even though some other studies reported higher frequency of timely seeking care [16, 25, 28] for fever. A study conducted in Tanzanian, 78% health facility visits took place within 24 hours of the onset of fever [28]. It was 51.3% in Ethiopia [16] and 40.3% in Senegal [25]. This could reflect that community’s prompt care seeking behavior has not improved as expected, and this could slow the move towards the national target of 100% treatment seeking within 24 hours after onset of fever [8].

It was found that factors such as irregular availability of community based health workers at health facilities and shortage and irregular availability of anti-malarial drugs at lower level of health facilities discouraged people from seeking prompt care. Malaria control program needs to take into account these constraints to ensure malaria commodities are regularly available as near to the community as possible. On the other hand, people’s perception and reaction to malaria illness is a crucial factor in early and appropriate care seeking. In this study, malaria was less perceived as severe disease, and as result, there was a tendency to wait until the fever turns to severe illness. This conveys the need to emphasize severity of malaria illness in malaria education program. Moreover, lack of resources such as money for transportation and other expenses impeded timely care seeking, especially for individuals who were far away from health facilities. This factor was more prominent given irregular availability of HEWs and shortage of anti-malarial drugs at health post as people are forced to visit the next higher level of care where demand for resource could increase. Since most malaria related death occurs within 48 hours, after onset of illness [31], the present finding communicates strong implications for the need to improve early diagnosis and early use of antimalarial medicines to reduce the burden of malaria in endemic areas.

This study revealed that formal health care facilities were sole sources of care sought for malaria illness which shows a drastic change in community behaviors on seeking care from appropriate sources. However, many previous studies reported diverse sources of care for malaria including herbal medicine, drug sellers and self-treatment [18, 20–22, 24, 25, 28]. It seems that most people had sufficient knowledge regarding appropriate treatment option for malaria, and as a result, seeking care from non-formal sources of care was non-existent. However, seeking spiritual support for severe illness and moving back and forth between traditional and formal sources of care cannot be ruled out for all families, as investigated in qualitative part of the current study. In Ethiopia, HEWs use RDT for malaria at community level and administer ACT [10, 12] which might have contributed to the increased awareness and care seeking behavior from formal sources of care.

With respect to use of any drug during the illness, about three-fourth used certain type of drug with less variability by age and sex. This figure is higher compared to some previous studies [18, 25, 28, 32]. Restricting the analysis only to those who received anti-malarial drugs, Chloroquine was the most frequently used drug followed by ACT. The use of Chloroquine might reflect that *Plasmodium vivax* is the predominant malaria parasite species which also reported in some studies from those localities [33,34]. Furthermore, given the distribution of ACT in private clinics is under restriction in most areas in Ethiopia, it might have influenced the private health care providers to go for chloroquine prescription which might have increased the frequency of reported Chloroquine use. In fact, the majority of those who used Chloroquine accessed it from private clinics (65.5%) and pharmacies (20.7%) where proper diagnostic facilities are poor which led into indiscriminate use of chloroquine for all types of fever and both types of *Plasmodium* species. Supporting evidence was documented in a multi-country study conducted in Africa [32]. However, this issue needs further investigation. Despite the restrictions of some anti-malarial drugs such as ACT at private health facilities, a significant proportion of individuals with febrile illness are still obtaining their medication, including ACT, from private health facilities which may indicate leakage of ACT from public to private facilities whereby affordability issue may be a concern. This entails the need to establish or strengthen public-private partnership for effective management of malaria. However, for indepth understanding of this issue, further studies are needed to investigate the flow of anti-malarial commodities between and within private and public health facilities particularly at low level health care systems.

Although anti-malarial drugs are distributed for free in public health facilities in Ethiopia, the majority of the respondents who got this medication reported that they paid for it. Given that private clinics and pharmacies (68.6%) were the major sources of anti-malarial drugs, the reported payment for this medication could be a logical finding. However, since some patients received drugs in addition to anti-malarial drugs, it is not possible to differentiate for which type of drugs they had been charged. Furthermore, health facilities might have charged patients for consultation and diagnosis and people might have considered that as payment for drugs which led to over-reporting of payment for anti-malaria drugs.

According to the Ethiopian Health Care delivery System [9,10], it is expected that febrile cases should be first report to health post/health extension workers. Contrary to this expectation, the majority of the febrile cases visited private clinics, pharmacies and health centers to seek treatment for fever or malaria bypassing the health post. This may need further investigation, and the health officials may need to review the function of the health extension program and the health posts in febrile case management.

This study showed that there was evidence of mis-use of anti-malarial drug (e.g. saving, sharing, discounting, self-medications, and doubling dosage). Given that malaria is an endemic disease in the area, people tended to save anti-malarial drugs for future use. It was noted that shortage and irregular availability/out of stock of anti-malarial drugs, and irregular availability of HEWs also contributed to inappropriate use of anti-malarial medications. On the other hand, community members were well aware of and very familiar with ant-malarial drugs, and this led to increased self-medications, and use of pharmacies, unregulated private clinics and medicines left over in homes as source of treatment at the onset of symptoms. These inappropriate uses of anti-malarial medicines are a potential threat to effective management of malaria and increasing concern for the development of drug resistance. This calls for promoting rational use of anti-malarial drugs for sustaining control and enhance elimination efforts.

## Limitations of the Study

The analysis of care seeking for fever was based on small cases with limited use of parametric tests. Nevertheless, it did not affect the precision of other estimates particularly knowledge and attitude. Furthermore, the sample size estimation was based on another outcome (i.e. long lasting insecticide treated net use). It should be noted that reporting of both illness episodes and treatment sought may be affected by incomplete recall of events. In addition, reported treatment sought might be intentionally misrepresented if use of certain source of treatment is perceived as more socially acceptable which might have resulted in underreporting of the care seeking from traditional sources. Further, the fact that this study was conducted during low malaria transmission season, the finding may not reflect year-round situations of care seeking for malaria in the area.

## Conclusions

Knowledge related to malaria was mainly moderate with quite high favorable attitude. However, there were some wrong beliefs and perceptions which might negatively impact desired malaria preventive behavior. The practice of care seeking for fever was quite high though the habit of seeking prompt care was very limited. Formal health care facilities were the sole source of care consulted for fever although private health facilities had also a significant share calling for the need to re-evaluate the role of private health facilities in the management of malaria for effective collaboration and partnership. However, further investigations are also required to understand the illegal leakage of some essential anti-malaria commodities from the public to the private sector. This might affect the cost of treatment and treatment seeking behavior in the poorest section of the community. Inappropriate use of anti-malarial drugs was prevalent in this community leaving a potential threat to effective management of malaria, drug resistance and hence the prospect of malaria elimination. Health care system factors, individuals’ perceptions and local beliefs contributed to delayed care seeking and inappropriate use of anti-malarial drugs. This demands evidence based and context specific behavioral change interventions while ensuring regular availability of malaria treatment services along with ensuring regular availability of frontline health workers. If the malaria elimination program does not take into account these factors, the progress towards optimal health seeking behavior might not be achieved in the near future.

## Supporting Information

S1 Dataset Analysis of knowledge and attitude related to malaria. (SAV)

S2 Dataset Analysis of fever prevalence among respondents. (SAV)

S3 Dataset Analysis of fever and seeking care. (SAV)

S1 File Background characteristics of respondents. (PDF)
